# Large Hyperplastic Colorectal Mass in a 25-Year-Old Intern Physician Suspicious for Inflammatory Cap Polyposis

**DOI:** 10.7759/cureus.87307

**Published:** 2025-07-04

**Authors:** Hunter T Pham

**Affiliations:** 1 Emergency Medicine, Michigan State University College of Human Medicine, Grand Rapids, USA; 2 Emergency Medicine, Corewell Health, Grand Rapids, USA

**Keywords:** cap polyposis, emotional distress in diagnosis, hyperplastic polyp, medically literate patient perspective, mucosal prolapse

## Abstract

This case study chronicles the identification and removal of a 40mm mobile, multilobulated, polypoid colorectal mass with ambiguous non-neoplastic inflammatory pathology. The initial pathology report identified “inflamed hyperplastic polyp with prolapse changes, ulcer, and granulation tissue,” consistent with the rare disease “inflammatory cap polyposis.” With few reported cases, cap polyposis is poorly described in the literature. In this case, colonoscopy did not identify additional masses, as is typical for cap polyposis, though a lone polyp does not exclude the diagnosis. The patient himself is the primary author of this case report, at the time of diagnosis, a 25-year-old male beginning his first year of emergency medicine residency. One year after excision, the patient underwent repeat colonoscopy, which demonstrated no recurrence of disease.

With the patient having the medical expertise to recognize that the large size of the polyp was concerning for neoplasm, there was significant emotional distress between the initial identification of the mass on colonoscopy and the results of the first pathology report five days later. Even with the first pathology report being negative for malignancy, there was only marginal emotional relief until receiving the results of the final pathology report of the wholly excised mass, 15 days after initial identification of the mass.

## Introduction

Colorectal polyps are a common finding in adults, with risk of neoplasia increasing alongside polyp size and patient age. Increasing rate of malignancy with mass size moves from 0% at <10mm, 0.9% at 10-19mm, 6.1% for 20-29mm, and 38.1% for >30mm [1]. Masses larger than 30mm are especially concerning, with either malignancy or high-grade dysplasia present in the vast majority of such lesions [1]. However, large non-neoplastic colorectal masses do occur and can pose diagnostic challenges, particularly when histopathology is ambiguous or suggests rare conditions.

Inflammatory cap polyposis is one such rare and poorly understood condition. Characterized by inflammatory polyps covered with a fibrinopurulent “cap,” the disease often presents with multiple lesions and symptoms such as mucous diarrhea and rectal bleeding. Since its initial description in 1985, fewer than 100 cases have been reported in the literature [2], and even fewer involve a solitary polyp [3,4]. The pathogenesis is not fully elucidated, though associations with mucosal prolapse and mechanical trauma have been proposed [2,5,6].

This case report describes the identification and management of a large, solitary rectal mass in a 25-year-old intern physician. The case is notable not only for the rare pathological findings but also for the emotional complexity faced by a medically literate patient navigating a potentially serious diagnosis at the onset of his medical career.

## Case presentation

As a 25-year-old male, the patient presented to his primary care provider with complaints of rectal bleeding and mucous stool for roughly six months. Additional symptoms included protrusion of a small amount of tissue during straining. There were no other symptoms including nausea, vomiting, appetite changes, weight changes, or changes to bowel movements. At that time, the patient had no prior medical history. He took only venlafaxine for generalized anxiety. Family history was remarkable only for colon cancer in the patient’s maternal grandmother, identified when she was 64 years old. The patient was monogamous with his wife and had no prior colonoscopies or anorectal trauma.

**Figure 1:**
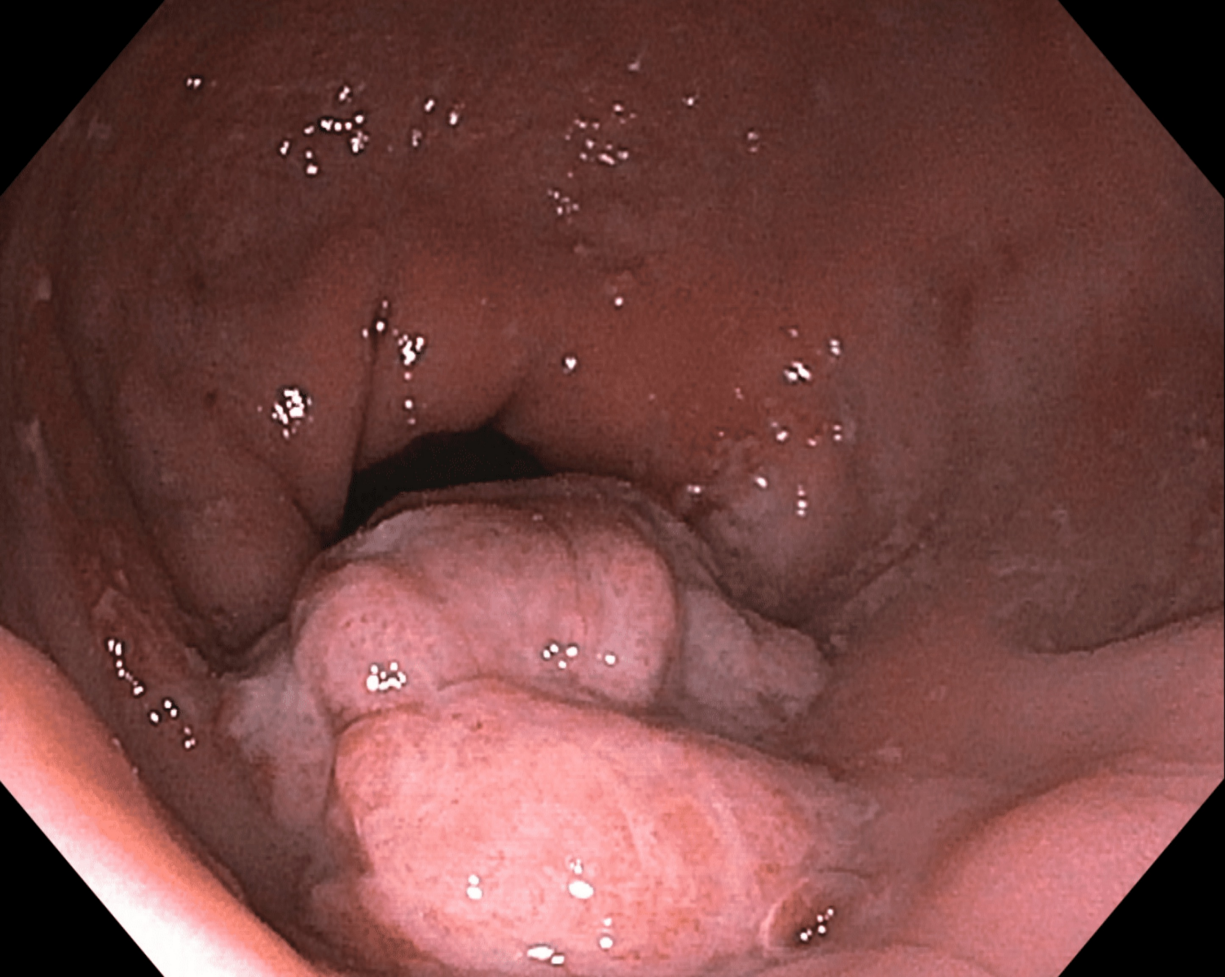
Rectal Polyp, View 1 Image of multilobulated polyp identified on diagnostic colonoscopy prior to surgical excision.

**Figure 2:**
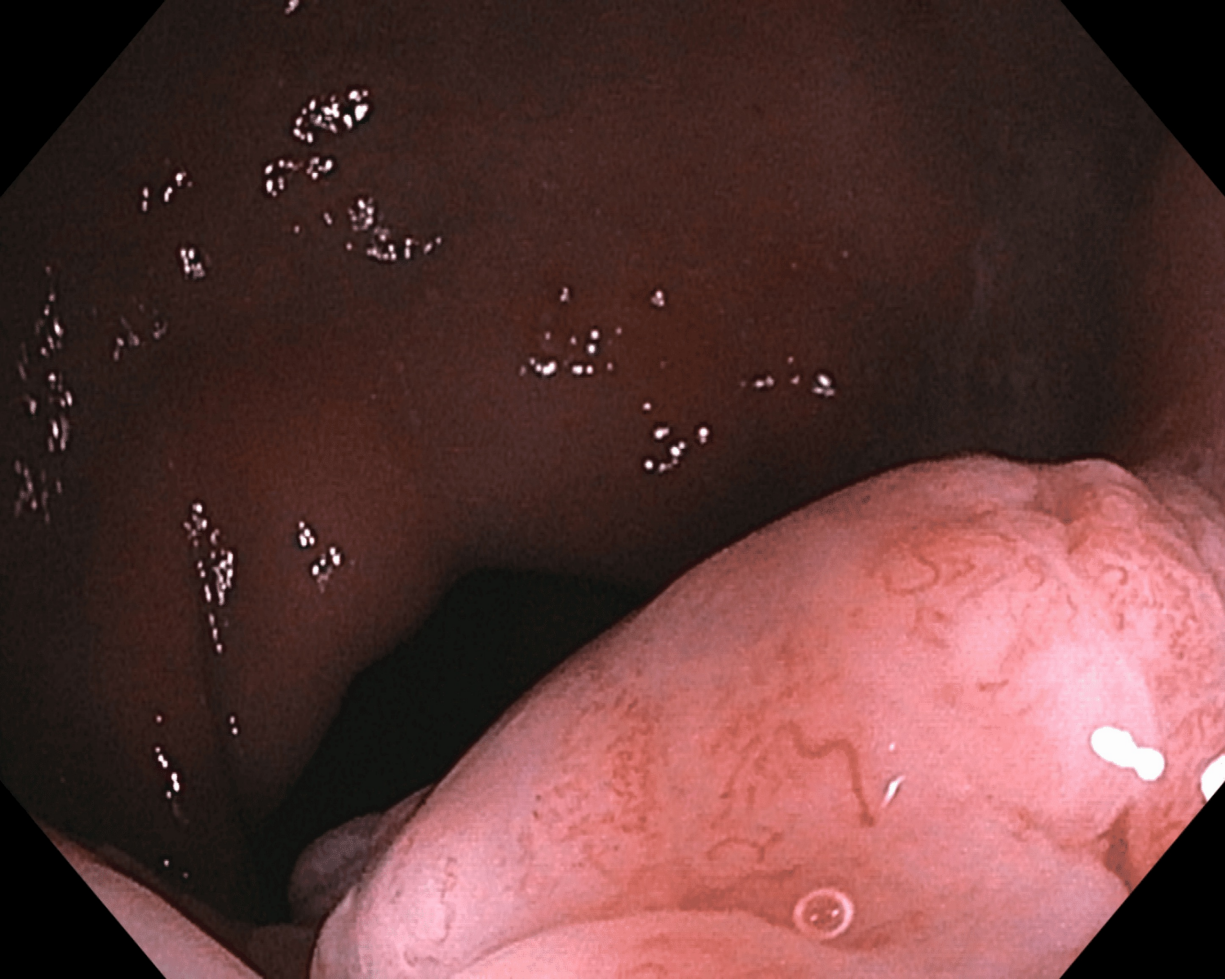
Rectal Polyp, View 2 Image of multilobulated polyp identified on diagnostic colonoscopy prior to surgical excision.

At the time of initial evaluation by the primary care provider, the patient refused an in-office examination, with both the patient and the provider sharing the assumption that the rectal bleeding and tissue protrusion were due to internal hemorrhoids. Colonoscopy was scheduled with the intention to band internal hemorrhoids. The colonoscopy revealed a 40mm mobile, multilobulated, polypoid mass (Figures 1, 2) in the rectum roughly 4cm from the anal verge. Hot snare debulking removed roughly half of the polyp, and a specimen was sent for histopathological analysis. Given the clear visualization of the lesion on colonoscopy, additional imaging was deferred.

The pathology report revealed “prolapse changes, ulcer, and granulation tissue” most consistent with inflammatory cap polyposis. Transanal excision of the mass was scheduled for 13 days after initial colonoscopy, with submucosal dissection of rectal tissue for total removal of mass. Presurgical laboratory testing in the form of complete blood count and comprehensive metabolic panel was completely within normal limits. No additional laboratory testing was performed. The pathology report of that excised tissue revealed “florid features of mucosal prolapse, ulceration, dense mixed inflammation, and reactive epithelial changes, negative for evidence of malignancy. The resection specimen demonstrates diffuse inflammatory changes and fibromuscular hyperplasia consistent with mucosal prolapse-related changes. Extensive overlying ulceration is seen with areas of dilated glands containing mucinous material/disrupted pools of acellular mucin. These mucinous areas are surrounded by lamina propria and show no evidence of dysplasia. These mucinous areas may be secondary to recent procedure site effect or may be part of the spectrum of mucosal prolapse (colitis cystica profunda).”

**Figure 3:**
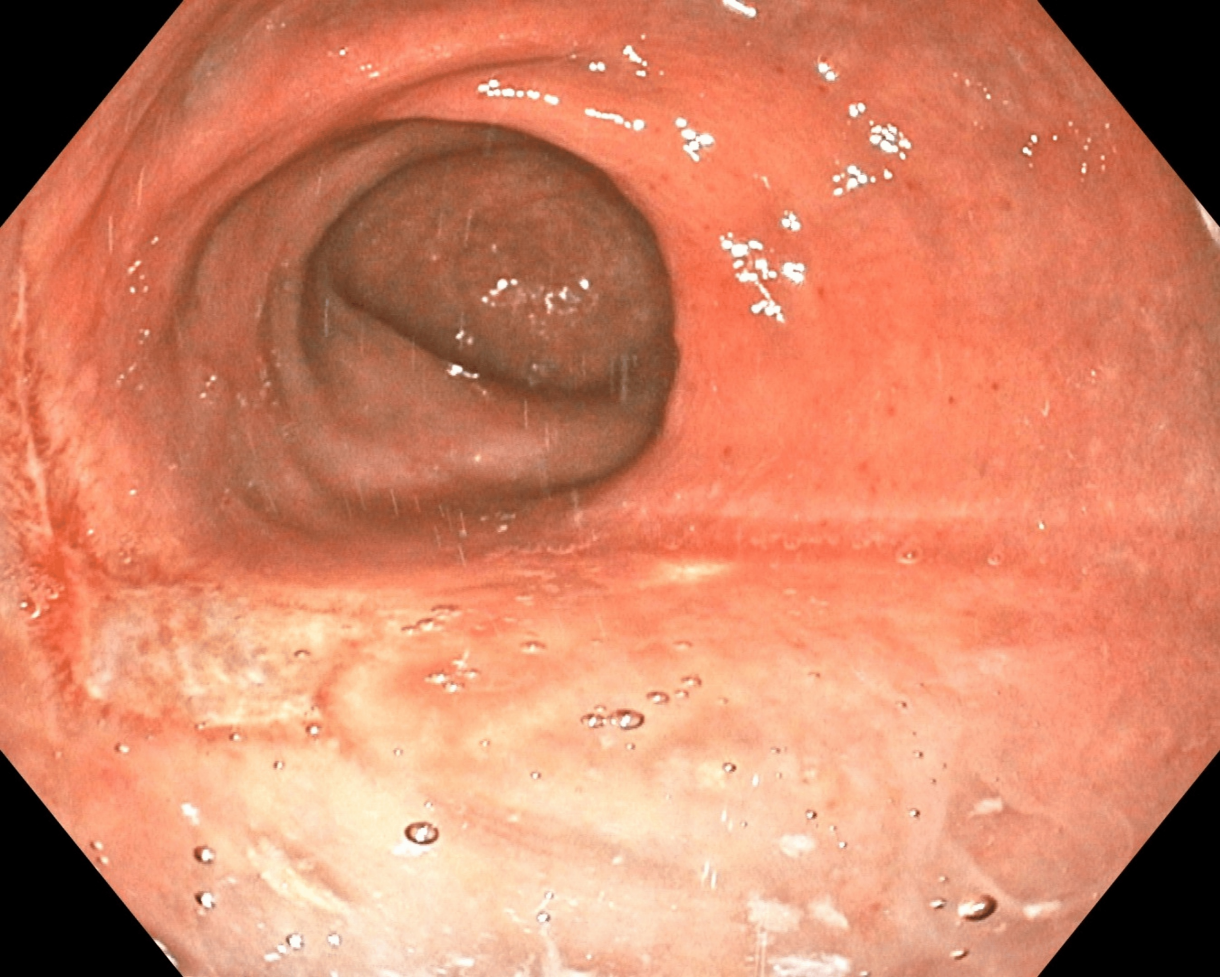
Postoperative Pyogenic Granuloma Image of pyogenic granuloma at the surgical site one year after transanal excision of multilobulated polyp.

The patient experienced an uneventful recovery with return to normal activity within one week. Following surgical excision, all symptoms resolved. Colonoscopy performed one year later demonstrated no recurrence of disease. Biopsy at that second colonoscopy was remarkable for pyogenic granuloma (Figure 3) at the site of operation, not warranting further investigation.

## Discussion

Such a large colorectal mass is certainly unusual in this patient’s age group. Masses greater than 30mm have a high association with malignancy, with around 90% being neoplastic [1]. Among those large neoplastic masses, almost half were identified as malignant, with the remainder of the recorded masses consisting of high-grade dysplasia. With the likelihood of malignancy clearly associated with size, one can infer that a mass of 40mm has an even higher likelihood of malignancy compared to 30mm, which was the highest measurement in this retrospective analysis [1].

The initial pathology report suggested an interesting possible diagnosis of inflammatory cap polyposis. For comparison, a typical histopathological description of inflammatory cap polyposis has been proposed: “Inflamed mucosa with tortuous, elongated crypts attenuated towards the mucosal surface with abundant inflammation in the lamina propria and characteristic ‘cap’ of inflamed and ulcerated granulation tissue on the mucosal surface” [7]. As alluded in the described patient’s pathology report, the inflamed hyperplastic polyps with prolapse changes, ulceration, and granulation tissue is consistent with literature descriptions of inflammatory cap polyposis [2]. Subsequent tissue pathology demonstrating “florid features of mucosal prolapse, ulceration, dense mixed inflammation” is not wholly inconsistent with cap polyposis. While more ambiguous than confirmatory in diagnosis, features of mucosal prolapse have been associated with cap polyposis [5,6], and mucosal prolapse is itself potentially a risk for developing cap polyposis [2]. The location of the polyp in this patient, as well as the presenting symptoms of rectal bleeding and mucous stool, are very typical for inflammatory cap polyposis [4].

While typically manifesting as multiple colorectal polyps [2], the diagnosis of inflammatory cap polyposis is not excluded because of a lone polyp [3,4]. Like most cases of a solitary polyp documented in the literature [4], the patient described in this case report had complete resolution of symptoms with polypectomy. While data is limited due to the condition’s rarity, three of nine reported cases of a solitary inflammatory cap polyp had complete resolution of symptoms with polypectomy, while those with more than one polyp required further surgical intervention [4]. There is some history of treatment of inflammatory cap polyposis with medications typical for inflammatory bowel disease like 5-aminosalicylic acid or sulfasalazine [4], but data on effectiveness is limited given the rarity of the diagnosis.

Beyond the medical intrigue of this case, there was a fair amount of emotional distress worth discussing. As previously mentioned, the patient described in this case is additionally the primary author of this case. As a recent medical school graduate at the time of evaluation, the patient had enough clinical knowledge to worry about the statistical likelihood of malignancy, despite the initial pathology report being reassuring. The colorectal surgeon did tell the patient that neoplasm could not be excluded, but that in his experience the external appearance of the mass was not consistent with neoplasm. Due to the size of the mass, the colorectal surgeon did label the pre-biopsy likelihood of neoplasm as “average” in medical records, which was immediately visible to the patient.

At 25 years old, having only recently graduated medical school and, at the time, finishing onboarding for residency, the patient experienced emotional distress at the concept of surgery and potential chemoradiation therapy. Especially prior to the initial biopsy findings, which took five days to obtain results, the patient admittedly perseverated over the effects of surgery and chemoradiation therapy on his residency career. Even after those results, some anxiety persisted over the possibility of a false-negative pathology report.

Fortunately, the patient’s residency program was both understanding and accommodating. The program director encouraged him that regardless of results, the patient’s residency training would proceed with as much assistance to the patient as needed. Following surgical excision of the mass, the residency program gave the patient fewer shifts for the scheduling block relative to his peers to allow for faster recovery. Emotional distress was relieved by the second pathology report of the whole mass, which was obtained 15 days after initial identification of the mass.

## Conclusions

This case underscores the diagnostic and emotional complexity surrounding large colorectal polyps in young adults. This 40mm rectal polyp ultimately demonstrated benign pathology potentially consistent with inflammatory cap polyposis, a rare and poorly understood condition. Beyond its clinical relevance, this case also highlights the unique emotional burden faced by medically literate patients navigating serious diagnostic concerns, particularly at critical junctures in their personal and professional development. With complete symptom resolution and no recurrence on follow-up colonoscopy, this case contributes to the growing body of literature on solitary inflammatory cap polyps and emphasizes the importance of holistic care that considers both clinical and emotional well-being.
